# Textual Practices in Crafting Bioethics Cases

**DOI:** 10.1007/s11673-012-9407-6

**Published:** 2012-11-01

**Authors:** Brian Hurwitz

**Affiliations:** Centre for the Humanities and Health, King’s College London, London, WC2R 2LS UK

**Keywords:** Case, Narrative, Bioethics, Composition, Interpretation

## Abstract

Bioethics case reports generally treat aspects of moral fathomability, characterised and addressed in different ways. This paper reads the case as a textual model of scenarios and draws attention to its structure, narrative shape, linguistic register, and the effects of tone and temporality on reader expectation and responsiveness. Such textual elements of case composition reflect authorial purpose and influence the interpretation, including moral and ethical interpretation, of bioethics cases.

The most interesting parts of the moral world have to be “read,” rendered, construed, glossed, elucidated, and not merely described (Walzer 1987, 29).

Ethical issues in health care have been represented and explored in a multiplicity of forms, including novels, dramas, short stories, memoirs, vignettes, and case reports (Rosenstand 2005; Singer and Singer 2005; Paola, Walker, and Nixon 2010). Factual, fictional, biographical, and clinico-literary case depictions have turned on conflicts of personal or public value, clashes of moral principle and commitment, and frustrated promises of resolution. Literature, broadly understood, provides readers and ethicists alike with rich representations of situations finely graded by moral ambiguity and viewpoint (MacIntyre 1981; Chambers 1996, 1999; Widdershoven and Smits 1996), and in this sense embodies and structures cultural encounters with notions of right and wrong (Guroian 1998).

In the preface to his novel, *What Maisie Knew* (1897), Henry James wrote that

the effort really to see and really to represent is no idle business in face of the constant force that makes for muddlement. The great thing is indeed that the muddled state too is one of the very sharpest of realities, that it also has colour and form and character, has often in fact a broad and rich comicality (James 1985, 30).

One way in which clinicians and ethicists respond to moral muddlement is by extracting what appears to be the salient elements of a situation—the medical and moral particulars—to incorporate them as observation, description, point of view, and components of ethical commentary into a text, woven together with narrative devices to create a case. Through devices such as retrospective reconstruction, controlled unfolding, the use of voice (witness, dialogue, and quotation), the development of suspense, surprise, and temporal switches, cases package situations in second-order formulations supposedly less quirky and particular than the phenomena they encapsulate (Holm 1997; Hurwitz 2005; Miller 1996).

Cases deposit representations of scenarios that can be explored and interrogated “on paper” so that they can be mused over, elaborated on, published, debated, and analysed, a process which distracts attention away from the lived experiences and perplexities of people in the grip of circumstances, by interposing critical distance between events and happenings themselves and the events narrated. The teller of cases reorders and reflects on situations (undertaking meaning-making a posteriori), exposing selected elements to the moral lens and rehearsed ethical reasonings of a wider community. In this sense, cases are “usable stories” (Scheiber 1979, 482), models informed by observation, selection, and ethical theory, through which perspective, moral deliberation, and commentary can be developed (Hurwitz 2006).

Composed of tellings that have a time course, a plot, a point, and a point of view (Charon 2006), case description occupies a diverse linguistic register—conversational, ethnographic, novelistic, anecdotal, naturalistic, impersonal, literary, and biographical. This paper claims a role for unpacking the textual form to illuminate how style in bioethics cases relates to authorial engagement with the moral situatedness of its subject matter (Parker 2012).

Lauren Berlant finds cases arise wherever and whenever problem-events animate judgements—a symptom, a crime or a moral quandary, “any irritating obstacle to clarity” in texts that develop a treatment of fathomability:

When an event occurs out of which a case is constructed it represents a situation in which people are compelled to take its history, seek out precedent, write its narratives, adjudicate claims about it, make a judgment, and file it somewhere: a sick body, a traffic accident, a phenomenon, instance, or detail that captures the interpretive eye. … [T]he idiom of the judgment … varies tremendously across disciplines, [and] professions: law, medicine, chat shows, blogs, [and] each domain [has] its vernacular (Berlant 2007, 663–672).

Case narratives manipulate temporality: The listener, spectator, or reader must imagine not only the events that “actually happened” but also the ways in which these are arranged in a telling. “Some literary critics call the former the ‘story’ and the latter the ‘plot.’ The plot construes the story in a certain way” (Hurwitz, Tapping, and Vickers 2006, 487), in a dual time-scheme arising from the difference in temporality between the signs (on a page or in speech) carrying meaning and the account narrated. In-and-of themselves, the signs making-up a narrative—its successive words, phrases, gestures, and images—lack storied temporality; it is in their concatenation and positioning of chronology that the plot takes shape (Rimmon-Kenan 2006). Hence Christian Metz’s formulation: that narratives work “by inventing one time sequence in terms of another time scheme” (quoted in Genette 1980, 33).

Bioethics cases constitute versatile means for *reporting that…* in various ways, voices, and emphases, allowing readers to *relate to…* and *assess* whatever is going on medically, interpersonally, psychologically, and morally. An ethics vignette reported by Chambers and Montgomery illustrates the freedom and responsibility exercised in the composition of cases, consideration of which may assist in their interpretation.

Suppose a colleague approaches you and says, “We have a patient who came for a prostatectomy, and as he was getting prepped, the nurses noticed that he wouldn’t talk about his family. Really odd. Then, after the surgery, he tells us that his wife and children—even his secretary—all think he’s away on a business trip, and he doesn’t want us to contact them. Now Mr. Kaufman’s bleeding, and we just took him back to the operating room” (Chambers and Montgomery 2002, 84).

This story, they argue, could be said to be the sequence of events:

(1) a man learns he needs surgery, (2) he tells his family he is away on a business trip, (3) after the surgery he tells the health care professionals about the deception, (4) he suffers a bleeding complication of surgery (Chambers and Montgomery 2002, 84).

The second version looks superficially similar to the original, but it is actually structured quite differently. Version one begins with the surgery and features a flashback observation that prior to surgery the patient would not talk about his family. In the first way the story is told the significance of Mr. Kaufman’s avoidance initially goes unnoticed; it is only recognised as significant retrospectively, emphasised by “he wouldn’t talk about his family,” which gains the status of a clue in an unfolding mystery and which causes some perplexity to health care staff (and here we might note that many clinical and bioethical cases are constructed on the template of a fathomable puzzle using techniques that highlight something odd at the start of a tale and, by a process of informational drip-feed, come to generate suspense not unlike that which takes place in detective fiction [Snyder 2004]).

As Chambers and Montgomery note, Mr. Kaufman has undergone a surgical procedure entirely alone, without the advice, support, or knowledge of family or a network of friends or colleagues. Does he harbour a secret (or secrets)? Is he abnormally solitary? Is it possible he wants to protect his family from worrying about him? Does he fear impotence as a consequence of the procedure, which he hasn’t discussed with his wife or partner? Is he living a double life? Such questions reveal how narratively-based are thoughts and hypotheses that come to the fore when someone’s behaviour appears strange or incomprehensible when explanations are sought to impute motive and bridge gaps in knowledge or understanding.

In the first way the story is told the account breaks off as the patient is bleeding and being taken back to the operating theatre, when the questions on the minds of his health carers, readers surmise, are not only “How serious has the situation become?”, “What’s to be done if the bleeding complication cannot be stopped by emergency surgery?”, and “Who, if anyone, should be informed about it?”, but additionally, and just as importantly, “What *sort* of a person is Mr. Kaufman?”, “What are his values, in what does he believe?”, and “What does he think he is doing?” Answers to such questions cannot be inferred without further biographical information, without, that is, knowing a great deal more about Mr. Kaufman’s personal story, which might reveal information about his life, lifestyle, character, deviousness, personality, goals, predilections, support group, mental health, upbringing, relations with parents, emotional ties, and loneliness (Hurwitz, Cushing, and Chisnall 2012).

But in the more linear way in which the story unfolds, what seems to be at stake is rather different, the focus revolving around a deception Mr. Kaufman has played on his family, which deemphasises the significance of his refusal to talk about family with health care staff. Chambers and Montgomery comment that, of the two ways in which the story has unfolded so far, one captures the urgency and existential drama of the situation, hinting at the effects on Mr. Kaufman’s health carers, their sense of discomfort, puzzlement, surprise, and possible disapproval of him: “he tells us that his wife and children—*even* his secretary—all think he’s away on a business trip” (Chambers and Montgomery 2002, 84, *emphasis added*), the phrase “he tells us” indicating that Mr. Kaufman’s reliability as a narrator may be doubted. However, the second telling of the case, based on more passive grammatical construction, does not index any issue about reliability, edits out the drama, and offers no reference to the health care staff who may well feel they are at the centre of an impending moral quandary.

So, the two case construals of Mr. Kaufman’s situation are by no means equivalent—their situational context and focus differ markedly. We might say that each case models the medico-moral scenario differently. In the first account, we learn of the existence of a wife, children, and a secretary, three sets of people with particular relationships to, and likely moral calls on, Mr. Kaufman, which his behaviour appears to be denying. In letting it be known he will not talk about his family, Mr. Kaufman is clearly demonstrating agency—operational autonomy—through which (for unknown reasons) he appears to have spun a web of deceit. In narrative terms, Mr. Kaufman is a character who is hard “to read”; in health care terms, he’s a person difficult to assess and understand. But in version two of the telling this context is omitted, presumably on the grounds that it is not morally salient.

Gilbert Ryle recognised that schematic descriptions of events and actions can be morally “thin.” The contraction of an eyelid may be an involuntary response to dust in the air, or it may be the signal a bidder employs to seal a contract at auction or the conspiratorial wink adopted to tell a confederate in a burglary to pull the trigger on an innocent victim. The meaning discerned in descriptions of events and actions—including moral meaning—depends critically on the context in which they are embedded: that the twitch of an eyelid can even be an action answering to a motive, expressing a certain desire, and not merely the reflexive consequence of irritation on the cornea, depends on just such a context, knowledge of which “*thick*ens” the moral meaning of a situation (Ryle 1971; Geertz 1993).

Fifteen years ago Barbara Nicholas and Grant Gillett made a plea for much more context in bioethics cases, complaining that “it is too frequently invisible within a method which seeks some sort of universal approach” (Nicholas and Gillett 1997, 295). In telling his family “he is away on a business trip” (version two), Mr. Kaufman’s action is portrayed in a morally thin way, whereas “he tells *us* that his wife and children—even his secretary—all think he’s away on a business trip” (Chambers and Montgomery 2002, 84, *emphasis added*) is a much thicker description, carrying more information about the scope of the deception and the kind of problem Mr. Kaufman’s health carers may face if he deteriorates further. The second version’s narrator, in contrast, is anonymous and disembodied and in no way affiliated with the health care staff responsible for Mr. Kaufman.

It is apparent how differentially health care tellings can highlight, place into shadow, and leave out the morally constitutive elements of a developing scenario. If the account of Mr. Kaufman’s post-operative bleeding were to feature in an organisational narrative about how well a hospital is (or is not) working, the case report might dwell on the fact that in version one the nurses, prior to surgery, did *in fact* notice that Mr. Kaufman would not speak about his family, but their observation somehow became dismissed and normalised—possibly by the nurses themselves—as not noteworthy until after the first surgery (Gabriel 2004). But if such an account were to feature in a medico-legal discussion about what happened, the narrator might focus on whether omitting to enquire further, at the pre-surgery stage, represented customary nursing practice and whether, in the circumstances, this could be considered reasonable; if it is not reasonable, could failure to perceive the significance of Mr. Kaufman’s silence amount to poor (even substandard) nursing practice? Is the nurse’s original observation and judgement revealed by the remark “*really odd*” (Chambers and Montgomery 2002, 84, *emphasis added*) documented in the nursing notes? And, if so, should it have been followed up? If not, why not? Or did “really odd” actually emanate from an interpolation of an authorial point of view into the telling of the case? We see here how one set of events—what happened when Mr. Kaufman underwent prostatic surgery—can be emplotted in a number of different ways depending on the purposes of recounting them (Hurwitz, Cushing, and Chisnall 2012; Mattingly 1998).

Narrative considered as discourse and discursive performance offers enormous scope for strategy and ingenuity in just *how* what is being narrated is constructed and conveyed, orally, gesturally, and as text. The framing and narration of a case—not only which elements are emphasised and which omitted but its narrative *structuring*—speaks in important ways to the purpose animating its telling and carries implications for how it is to be read and interpreted. It is not just a question of content, though that is critical, but of the *shaping, accenting*, and *structuring* of informational content (Hurwitz, Cushing, and Chisnall 2012). As with the case of Mr. Kaufman, the plot’s construal needs to be read in part as the outcome of narratorial choices. Building the case and composing its narrative are features of bioethics cases that deserve as much attention as does case analysis, where case *qua* model or construct is often treated as a given.

However, cases are not in any meaningful sense “given”; rather, they are narrative construals of observations and ways of thinking-through representations. They are not photo-mechanical reproductions of scenes on a stage but artifices of theory-informed thought, albeit frequently rendered in depersonalised, “naturalistic” language that mimics the medical literatures of fact (Chambers 1996, 1999). Chambers has argued for the *absence* of privileged and innocent compositions of ethical problems and has urged that what is required for their analysis is a sophisticated response to the “constructedness” of their composition, which often “makes readers see another’s way of seeing as natural and self-evident” when it may not be (Chambers 1999, 25–30).

Martin classifies case descriptions by the schema to which they approximate (Martin 2008): A running commentary on a problem that never develops to a critical nub he terms a “recount”; whereas an “exemplum” explicates a noteworthy incident and encourages moral judgement on the part of the reader; and an “anecdote” develops a scenario that disrupts in some way the reader’s expectation, encouraging an act of sympathy or identification with a character or action, such as “there but for the grace of God go I.”

Some case genres are better suited to delineating complex moral issues than others, at accommodating in-depth development and exploration of a scenario, and in evoking reader responses. Identifying the genre in which a case is written goes some way to indicating how it is constructed and to pinpointing how it works on readers. A case published by the *British Journal of General Practice* in 2006 is clearly an exemplum in Martin’s sense, one in which it is the reader’s response that actually supplies the moral closure to the story:

Mrs B was 84 years old, when her General Practitioner, who had known her for a decade and a half, was asked to see her. Mrs B had been widowed for 5 years, following the sudden death of her husband, Jack. Her two sons had been a disappointment to her: both were in and out of prison for repeated minor criminal offences. The practice nurse had asked the doctor to see her, after looking at her blood test results. Below we show Mrs B’s multiple and compounding conditions, and the results of her most recent tests, which sparked the consultation.

**Table:** 

Mrs B’s diagnoses	Lab test results
Diabetes	HbA1c 9.7 %
Hypertension	BP 180/96
Osteoarthritis	BMI 29
Macular degeneration	Cholesterol 8 mmol/L
Depression

The doctor rehearsed with Mrs B the abundant evidence supporting interventions to improve all her biochemical parameters. There was evidence, the doctor said confidently, to support changes in her lifestyle. Mrs B listened carefully to the doctor, and then remained quiet for a moment. After a while, she spoke. “Well”, she said, “Jack’s dead, and the boys have gone” (Sweeney and Heath 2006, 386).

The most prominent aspect of this report, which somewhat curiously is laid-out in the format of a medical record, is the way it suddenly comes to an abrupt halt, just as it transpires that patient and doctor inhabit worlds of radically different concerns and values. The “abundant” evidence (and confidence) with which we expect the doctor to assess Mrs. B takes on a new complexion as a result of the words she utters. What Mrs. B says (and the way that she speaks) makes clear that all the medical evidence in the world is of no significance to her, because she has quite different things on her mind, the tone, tense, and terseness of her utterance lending pungency and poignancy to her words. Perhaps what jars the most in the reading of this case is the glimpse it provides into Mrs. B’s inner world, the intensity of her distress and sadness, which breaks out on an otherwise apparently tranquil consultation, the story ending quite abruptly “in mid-air.”

What Mrs. B most needs, readers will surmise, is a doctor who is not totally in thrall to bio-variables, one who can switch his or her attention to just how lonely and hopeless this widow feels, a response which acts as the moral closure to the case (Hurwitz, Cushing, and Chisnall 2012).

The way cases work requires unpacking within the parameters of their adopted composition and stylistic convention. A common strategy is for a case to masquerade as a mirror image of a situation under scrutiny:

AIDS and a Duty to Protect

Mr B, age twenty-eight, reported to the community health center of a large city teaching hospital after being confidentially informed that his blood test was positive for antibodies to HIV, the virus that causes AIDS. The patient had no symptoms.

Dr T informed Mr B that although he did not have AIDS, there was between a five and thirty-five percent probability that he would develop the disease within the next five years. He was told that he could probably infect others through sexual contact, by sharing needles, or by donating blood and blood products. He was counselled not to donate blood, and to engage in “safe sex”, that is, sex that does not involve the exchange of bodily fluids such as semen.

Mr B then revealed that he is bisexual, and that he believed that he had contracted the infection during one of his homosexual encounters. He also said that he was engaged. Dr T advised him to inform his fiancée of his diagnosis. But Mr B refused to do so, saying that it would ruin his marriage plans.

Should Mr T inform the fiancée of Mr B’s test results, or should he protect the confidentiality of the therapeutic relationship? (Crigger 1998, 43).

The impersonal register here is very striking: Rendered in a thoroughly disinterested style, this case unfolds in a linear manner, offering no reference at all to human interiority—voice, feeling, the personality of patient or doctor. It is therefore not possible to begin to explain the patient’s adamant refusal to inform his fiancée of his HIV status. This way of writing removes the subjective reality of difficulties faced by the *dramatis personae* of a case: what Mr. B may feel, how he reasons, and how the doctor intends to respond to his point-blank refusal. We learn nothing of the doctor’s appreciation of Mr. B’s sense of moral and personal responsibility: Is Dr. T considering whether B is scared on his own behalf, or is he more afraid that his fiancée will break off the engagement when she hears about the HIV status or about his bisexuality? And does Dr. T have any inkling of how Mr. B responded on first hearing of his HIV test result? Does the doctor appreciate that people need time, space, and opportunities to converse with others in order to come to terms with news of this import? At what point should Dr. T try to explore Mr. B’s conscience concerning his feelings for—not only his moral responsibility toward—his fiancée? About these interactional issues that carry much moral import the account is silent. If ethical and clinical progress is to be made in this scenario, some situated plan needs to be developed that can point toward a negotiated way forward, but there is no indication in the case that this could be attempted. These aspects of the situation are not the focus of the report, which instead confronts the reader with a clash of moral obligations, prefigured in a title strongly suggesting which one should triumph.

Clinico-ethical case descriptions neither match transcriptions of medical records nor the way health carers talk to patients, and although many medico-moral cases arise out of networks of human relations, the way they are written-up often occludes these interpersonal origins in favour of attempts to subsume the issues they raise under a discussion of universalist ethics, rather than within a more situated form of deliberation (Parker 2012). This has led some ethnographers and social scientists to propose that more empirical, anthropological, and participant observer methods be adopted to ensure bioethics cases and considerations are based upon validly collected data that authentically represent the local networks from which these texts arise (Kleinman 1997, 1999).

This paper has argued that understanding medico-ethical case reports can be enhanced by greater attention to their construction as models of health care events and happenings. Peter and Renata Singer, in an anthology of ethics entitled *The Moral of the Story* (2005), argue that literary accounts invoke layered depths of detail, moral ambiguity, richly nuanced characters, and circumstances that are recognised by ethical analysis of such works. Martha Nussbaum has noted something similar: that the language of philosophical cases often lacks the particularity, emotive appeal, and absorbing emplottedness of a good story—“fiction’s way of making the reader a participant and a friend”—that how tales are couched provokes different ethical responses and interpretations (Nussbaum 1990, 19 and 46).

Case writings influence readers through structure and affect as well as contents. In the clinic, techniques of “attentive listening” seek to take account of the shape and tone of consultations—the word order used, emotional intonation, pauses, and how silence and non-verbal utterances are deployed—in attempts to understand what is being communicated (von Fragstein et al. 2008). Close readings of texts, of the sort sketched out here, draw attention to the craft of case construction and the sorts of insights that can accrue from examining structure, effects on readers, and the textual practices that take place within the envelope of bioethics cases.
